# Relationships of rapid eating with visceral and subcutaneous fat mass and plasma adiponectin concentration

**DOI:** 10.1038/s41598-023-38623-7

**Published:** 2023-07-17

**Authors:** Hideki Tsumura, Mari Fukuda, Takashi Hisamatsu, Rie Sato, Rina Tsuchie, Hideyuki Kanda

**Affiliations:** 1 Graduate School of Technology, Industrial and Social Sciences, Tokushima University, 1-1, Minamijosanjima-cho, Tokushima-shi, Tokushima 770-8502 Japan; 2 Department of Public Health, Okayama University Graduate School of Medicine, Dentistry, and Pharmaceutical Sciences, 2-5-1, Shikata-cho, Kita-ku, Okayama-shi, Okayama 700-8558 Japan; 3 Department of Emergency and Critical Care Medicine, Faculty of Medicine, Shimane University, 89-1, Enya-cho, Izumo-shi, Izumo 693-0021 Japan; 4 Department of Nursing, Faculty of Medicine, Shimane University, 89-1, Enya-cho, Izumo-shi, Shimane 693-8501 Japan

**Keywords:** Weight management, Metabolic disorders, Nutrition

## Abstract

Rapid eating has been demonstrated to be associated with obesity and overweight. However, few studies have characterized the separate relationships of eating speed with visceral and subcutaneous fat mass or circulating adiponectin concentration. We hypothesized that rapid eating is associated with the larger visceral fat tissue (VFT) area and lower adiponectin concentration, but not with the subcutaneous fat tissue (SFT) area in men and women. We performed a cross-sectional study of 712 adults aged 20–86 years (528 men and 184 women; mean ± SD age 59.36 ± 13.61 years). The participants completed a self-reported questionnaire, and underwent anthropometric and laboratory measurements and computed tomographic imaging of the abdomen as a part of annual medical check-ups. Multivariate linear regression analyses revealed that rapid eating was associated with larger visceral (B = 24.74; 95% CI 8.87–40.61, p = 0.002) and subcutaneous fat areas (B = 31.31; 95% CI 12.23–50.38, p = 0.001), lower adiponectin concentration (B =  − 2.92; 95% CI − 4.39– − 1.46, p < 0.001), higher body mass index (BMI) (B = 2.13; 95% CI 1.02–3.25, p < 0.001), and larger waist circumference (B = 5.23; 95% CI 2.16–8.30, p < 0.001) in men, which is partially consistent with the hypothesis. In contrast, rapid eating was found to be associated only with BMI, and not with abdominal adipose area or adiponectin concentration in women, which is a result that is not consistent with the hypothesis. These results suggest that there is no difference in the association of rapid eating with VFT and SFT areas.

## Introduction

Obesity and overweight are defined as abnormal or excessive fat accumulation that poses a risk to health. According to the World Health Organization criteria, overweight is characterized by a body mass index (BMI) of ≥ 25 kg/m^2^ and obesity by a BMI of ≥ 30 kg/m^2^. Approximately 52% of adults aged ≥ 18 years had overweight or obesity^1^. Obesity and overweight are known risk factors for cardiovascular diseases, type 2 diabetes, and musculoskeletal disorders^1^.

Eating behavior is one of the contributors to obesity and overweight. A meta-analysis demonstrated that rapid eating, the consumption of a larger-than-normal amount of food per unit time, is associated with obesity and overweight^2^. The results were derived from community studies^3,4^ and from studies of patients with diabetes and hyperlipidemia^5,6^. This relationship was also identified in children and adolescents^7,8^. Although most of these findings were made in Asian countries, others conducted in non-Asian countries have generated similar results^9,10^. Longitudinal studies have demonstrated that rapid eating is predictive of future obesity and overweight^11,12^, and interventions aimed at reducing eating speed have been shown to reduce BMI in adults with obesity^13^. Taken together, these results suggest that rapid eating causes obesity and overweight.

In most previous studies, anthropometric techniques have been used to assess abdominal fat mass, including BMI, waist circumference (WC), and waist-to-hip ratio (WHR). While these anthropometric techniques are readily performed, they are not particularly accurate, specific, or reproducible measures of abdominal fat mass^14^. In addition, they are incapable of differentiating the visceral fat tissue (VFT) located around the abdominal viscera, in the mesentery and omentum, and the subcutaneous fat tissue (SFT) situated under the skin.

VFT and SAT have distinct lipid storage and metabolic properties. In particular, VFT mass is more predictive of developing cardiovascular disease, type 2 diabetes, and hyperlipidemia^15,16^. Computed tomography (CT) and magnetic resonance imaging (MRI) are the gold-standard methods of assessing abdominal fat mass, because they provide highly accurate and specific quantitative data and can be used to measure the VFT and SFT areas separately^14^. However, CT and MRI have been used to evaluate the relationships between eating speed and abdominal fat components in few studies. Previous CT and MRI-based studies have shown that rapid eating is associated with a larger VFT area but no difference in SFT area in non-obese adults^17^, apparently healthy adult men^18^, or children^19^. To the best of our knowledge, the relationships of eating speed with the VFT and SFT areas in both adult men and women have only been assessed in one previous study^17^ using CT or MRI. Because sex-differences in relevant parameters, including eating speed, metabolism, and visceral and subcutaneous fat distribution, have been found, further studies are necessary to confirm whether rapid eating is only associated with VFT, regardless of sex.

Adipocytes are thought to represent an endocrine organ because they secrete a large number of bioactive compounds, referred to as adipokines. Adiponectin is one of these adipokines; is mostly secreted by white adipose tissue; has insulin sensitizing, anti-inflammatory, and anti-arteriosclerotic effects; and is thought to protect against the development of metabolic syndrome and cardiometabolic diseases^20,21^. Adiponectin synthesis and secretion decrease with adipose hypertrophy, and lower adiponectin concentrations are associated with obesity and overweight^22^. Given that rapid eating is associated with obesity and overweight, it is also likely to be associated with lower adiponectin concentration. A previous laboratory study demonstrated that higher postprandial adiponectin concentrations are associated with rapid eating, rather than with slower eating^23^. However, to our knowledge, no studies have yet focused on the relationship between long-term habitual eating speed and circulating adiponectin concentration.

In the present study, we aimed to evaluate the relationships of eating speed with VFT, SFT, and adiponectin concentration in adult men and women. On the basis of the results of previous studies that showed relationships of abdominal fat mass and adiponectin with rapid eating^3,23^, we hypothesized that rapid eating is associated with larger VFT area and lower adiponectin concentration, but not with SFT area in men and women.

## Methods

### Study sample and protocol

We performed a cross-sectional study of 712 adults aged 20–86 years (528 men and 184 women, mean ± SD age 59.36 ± 13.61 years) who underwent annual medical check-ups provided by the Health Promotion and Health Check-up Center, JA-Shimane, in Shimane Prefecture, Japan. The participants comprised agricultural workers, employees of agriculture-related companies, and their families. They each completed a questionnaire and the medical check-up staff checked the submitted questionnaires and asked the participants to complete any incomplete items. Well-trained medical staff performed anthropometric measurements, blood sample collection, and CT imaging of the abdomen for each participant as part of their medical check-up.

The study was conducted according to the principles of the Declaration of Helsinki. The study was approved by the Medical Research Ethics Committee, Shimane University Faculty of Medicine (No. 20150630-1, approved on July 10, 2015). All the participants received detailed information about the study and provided their written informed consent before participation.

### Self-reported questionnaire

The self-reported questionnaire was composed of items regarding demographics (age and sex), lifestyle factors, and depression. Eating speed was rated on a 3-point Likert scale as “rapid”, “moderate”, or “slow” in response to the question “How do you eat in comparison to others?” The validity of this question had been demonstrated by the substantial agreements of self-reported eating speed with objectively measured eating speed^24,25^ and friend-reported eating speed^26^. This question has been shown to yield repeatable outcomes for 1 year^27^. With respect to lifestyle parameters that are thought to be associated with obesity and overweight, current alcohol consumption frequency (every day, sometimes, or rarely), current smoking (yes or no), skipping breakfast three or more times a week (yes or no), eating snacks after dinner three or more times a week (yes or no), having dinner within 2 h of bedtime three or more times a week (yes or no), and engaging in habitual physical activity (yes or no) were assessed. Depression was assessed using a two-item screen^28^. Respondents answered either “yes” or no” in response to the two questions, “During the past month, have you often been bothered by feeling down, depressed, or hopeless?” and “During the past month, have you often been bothered by having little interest or pleasure in doing things?”. A positive response to both items was considered to indicate possible depression.

### Physical and laboratory examinations

Height (in cm) and body mass (in kg) were measured using portable stadiometers, with the participants not wearing shoes, to the nearest 0.1 cm and 0.1 kg, respectively. BMI was calculated as body mass in kg divided by height in m, squared. WC was measured using a tape measure to the nearest 0.1 cm at the level of the umbilicus while the participants were in a standing position. Venous blood samples were collected in the morning after an overnight fast of at least 10 h. The plasma concentration of adiponectin was measured using a latex particle-enhanced turbidimetric immunoassay and an automated analyzer (Adiponectin Latex Kit, Otsuka Pharmaceutical Co., Ltd., Tokyo, Japan). The inter-assay coefficient of variation was < 10.0%, and tests of stored specimens have shown no biological degradation, indicating that the measurements were highly valid. The serum concentrations of low-density lipoprotein (LDL)-cholesterol, high-density lipoprotein (HDL)-cholesterol, and triglyceride were measured using enzymatic assay kits (Kyowa Chemical, Japan) and an Autoanalyzer 7350 (Hitachi Ltd., Tokyo, Japan). The VFT and SFT areas were measured at the level of the umbilicus using a car-mounted multi-slice CT scanner (ELCOS, Hitachi Ltd., Ibaraki, Japan) and specific software for the measurement of abdominal fat area (Hitachi Ltd.).

### Statistical analysis

Descriptive data are presented according to sex as mean and standard deviation (SD) for continuous data and percentage and frequency for categorical data. Continuous datasets were compared using one-way between-subject analysis of variance, followed by *post-hoc* pairwise comparisons using the Holm method; and categorical datasets were compared using Fisher’s exact test. To evaluate the relationships of eating speed with the SFT and VFT areas and adiponectin concentration, multivariate linear regression analysis, involving a forced entry method, was conducted, with adjustment for age, the frequency of current alcohol consumption, current smoking, skipping breakfast, eating snacks, having dinner within 2 h of bedtime, habitual physical activity, and depression. In addition, we evaluated the relationships of eating speed with BMI and WC using multivariate linear regression analysis and the forced entry method, adjusting for the covariates listed above. Variance inflation factors (VIFs) were computed for the relationships between explanatory variables to check for multicollinearity. All the analyses were conducted separately for men and women. Because the data were obtained from all the participants in annual medical check-ups, a sample size calculation was not performed. Two-sided *p* values < 0.05 were regarded as statistically significant. All the analyses were conducted using R (version 4.2.1; R Foundation for Statistical Computing, Vienna, Austria)^29^.

## Results

### Eating speed

The characteristics of the participants, categorized according to sex and eating speed, are shown in Table 1. Among the men, 161 (30.5%) had a rapid eating speed, 326 (61.7%) had a moderate eating speed, and 41 (7.8%) had a slow eating speed. Among the women, 42 (22.8%) had a rapid eating speed, 128 (70%) had a moderate eating speed, and 14 (7.6%) had a slow eating speed.

**Table 1:** Participant characteristics according to sex and eating speed.

Variables	Men								Women							
	Rapid eating		Moderate eating		Slow eating		F	p value	Rapid eating		Moderate eating		Slow eating		F	p value
Age (years)	54.81	(15.28)	61.01	(13.06)	61.15	(15.57)	11.07	< 0.001^a,b^	58.48	(11.09)	59.75	(12.04)	65.29	(8.46)	1.83	0.163
BMI (kg/m^2^)	24.30	(3.73)	23.17	(3.03)	21.98	(2.84)	10.87	< 0.001^a,b^	24.62	(3.45)	22.27	(3.34)	22.28	(2.48)	8.19	< 0.001^a,b^
WC (cm)	83.51	(9.69)	80.64	(8.46)	78.23	(8.61)	8.34	< 0.001^a,b^	81.89	(9.39)	77.70	(9.44)	76.19	(8.77)	3.62	0.029^a^
VFT (cm^2^)	87.31	(50.57)	81.95	(45.88)	69.19	(52.11)	2.42	0.090	65.08	(31.65)	56.22	(33.70)	55.67	(20.98)	1.22	0.297
SFT (cm^2^)	122.01	(63.21)	103.04	(52.71)	85.44	(47.31)	9.71	< 0.001^a,b^	175.51	(73.23)	147.13	(70.30)	126.97	(57.62)	3.56	0.031
Adiponectin (µg/mL)	8.11	(3.70)	9.21	(4.68)	11.16	(4.54)	8.67	< 0.001^a,b^	13.68	(7.76)	14.92	(7.31)	17.84	(7.83)	1.65	0.195
LDL-cholesterol (mg/dL)	124.24	(30.13)	125.80	(29.10)	119.07	(35.42)	0.96	0.383	125.17	(27.15)	129.80	(31.79)	143.21	(44.77)	1.68	0.190
HDL-cholesterol (mg/dL)	58.24	(14.54)	60.38	(14.28)	67.95	(15.01)	7.43	< 0.001^b,c^	67.55	(13.97)	67.63	(14.25)	72.14	(17.52)	0.64	0.190
Triglyceride (mg/dL)	122.52	(85.22)	114.06	(80.81)	115.78	(77.68)	0.58	0.561	100.00	(58.87)	87.71	(41.64)	98.00	(48.72)	1.26	0.287
Frequency of alcohol consumption, n (% yes)							N/A	0.076							N/A	0.109
Every day	82	(50.9)	192	(58.9)	28	(68.3)			5	(11.9)	11	(8.6)	0	(0.0)		
Sometimes	46	(28.6)	64	(19.6)	9	(22.0)			6	(14.3)	43	(33.6)	4	(28.6)		
Rarely	33	(20.5)	70	(21.5)	4	(9.8)			31	(73.8)	74	(57.8)	10	(71.4)		
Current smoker, n (% yes)	45	(28.0)	105	(32.2)	15	(36.6)	N/A	0.456	2	(4.8)	8	(6.3)	0	(0.0)	N/A	1
Skipping breakfast, n (% yes)	22	(13.7)	34	(10.4)	2	(4.9)	N/A	0.256	3	(7.1)	12	(9.4)	1	(7.1)	N/A	1
Eating snacks, n (% yes)	29	(18.0)	41	(12.6)	6	(14.6)	N/A	0.262	6	(14.3)	21	(16.4)	3	(21.4)	N/A	0.845
Having dinner within 2 h of bedtime, n (% yes)	64	(39.8)	110	(33.7)	12	(29.3)	N/A	0.316	12	(28.6)	20	(15.6)	4	(28.6)	N/A	0.110
Habitual physical activity, n (% yes)	36	(22.4)	73	(22.4)	6	(14.6)	N/A	0.559	6	(14.3)	23	(18.0)	2	(14.3)	N/A	0.897
Depression, n (% yes)	12	(7.5)	24	(7.4)	4	(9.8)	N/A	0.783	7	(16.7)	12	(9.3)	1	(7.1)	N/A	0.419

Values are means (SD) or number (percentage).

*BMI* body mass index, *HDL* high-density lipoprotein, *LDL* low-density lipoprotein, *SFT* subcutaneous fat tissue, *VFT* visceral fat tissue, *WC* waist circumference. *Post-hoc* analysis: ^a^Significant difference between rapid and moderate eating, ^b^Significant difference between rapid and slow eating, ^c^Significant difference between moderate and slow eating.

### VFT and SFT areas

The results of the multivariate linear regression analyses for the VFT and SFT areas are shown in Table 2. For the men, the models for VFT area (F(11, 516) = 6.32, p < 0.001, adjusted R^2^ = 0.100), SFT area (F(11, 516) = 4.52, p < 0.001, adjusted R^2^ = 0.068), and adiponectin concentration (F(11, 516) = 6.96, p < 0.001, adjusted R^2^ = 0.111) were significant. Rapid eating was significantly associated with larger VFT and SFT areas, and rapid and moderate eating speeds were significantly associated with lower adiponectin concentration, after adjustment for age, the frequency of alcohol consumption, current smoking status, skipping breakfast, having snacks, having dinner within 2 h of bedtime, habitual physical activity, and depression.

**Table 2:** Regression models for visceral and subcutaneous fat areas and adiponectin concentration by sex.

Eating speed	VFT					SFT					Adiponectin				
	B (95% CI)		SE B	t	p value	B (95% CI)		SE B	t	p value	B (95% CI)		SE B	t	p value
Men															
Rapid	24.74	(8.87–40.61)	8.08	3.06	0.002	31.31	(12.23–50.38)	9.71	3.23	0.001	− 2.92	(− 4.39– − 1.46)	0.75	− 3.91	< 0.001
Moderate	14.49	(− 0.43–29.41)	7.59	1.91	0.057	17.07	(− 0.86–34.99)	9.12	1.87	0.062	− 2.18	(− 3.56– − 0.80)	0.70	− 3.11	0.002
Slow	0.00	(Reference)				0.00	(Reference)				0.00	(Reference)			
Women															
Rapid	14.41	(− 4.79–33.61)	9.73	1.48	0.140	40.94	(− 2.89–84.77)	22.20	1.84	0.067	− 2.33	(− 6.77–2.11)	2.25	− 1.04	0.302
Moderate	5.65	(− 11.72–23.03)	8.80	0.64	0.522	19.25	(− 20.41–58.90)	20.09	0.96	0.339	− 1.66	(− 5.68–2.36)	2.04	− 0.81	0.417
Slow	0.00	(Reference)				0.00	(Reference)				0.00	(Reference)			

Adjusted for age, the frequency of current alcohol consumption, current smoking, skipping breakfast, eating snacks, having dinner within 2 h of bedtime, physical activity, and depression.

*CI* confidence interval, *SFT* subcutaneous fat tissue, *VFT* visceral fat tissue.

For the women, the models for VFT area (F(11, 172) = 2.93, p = 0.001, adjusted R^2^ = 0.104) and adiponectin concentration (F(11, 172) = 2.69, p = 0.003, adjusted R^2^ = 0.092) were significant, whereas the model for SFT area was not (F(11, 172) = 1.40, p = 0.176, adjusted R^2^ = 0.024). Eating speed was not significantly associated with the VFT or SFT areas or adiponectin concentration after adjustment for the listed covariates. VIFs for the explanatory variables of 1.01 to 1.14 were obtained for all the regression models, which implies the absence of substantial multicollinearity.

### Anthropometric data

The results of the multivariate linear regression analyses for BMI and WC are shown in Table 3. For men, the models for BMI (F(11, 516) = 4.11, p < 0.001, adjusted R^2^ = 0.061) and WC (F(11, 516) = 3.03, p < 0.001, adjusted R^2^ = 0.041) were significant. Rapid and moderate eating was significantly associated with higher BMI, and rapid eating speeds were significantly associated with larger WC, after adjustment for the listed covariates. For women, the model for BMI was significant (F(11, 172) = 2.16, p = 0.019, adjusted R^2^ = 0.065), whereas the model for WC was not (F(11, 172) = 1.39, p = 0.179, adjusted R^2^ = 0.023). Rapid eating speed was significantly associated with higher BMI, but eating speed was not significantly associated with WC, after adjustment for the covariates. We obtained VIFs for the explanatory variables of 1.01 to 1.16 in all the regression models, which implies the absence of substantial multicollinearity.

**Table 3:** Regression models for body mass index and waist circumference by sex.

Eating speed	BMI					WC				
	B (95% CI)		SE B	t	p value	B (95% CI)		SE B	t	p value
Men										
Rapid	2.13	(1.02–3.25)	0.57	3.75	< 0.001	5.23	(2.16–8.30)	1.56	3.35	< 0.001
Moderate	1.18	(0.13–2.23)	0.53	2.20	0.028	2.49	(− 0.39–5.38)	1.47	1.70	0.090
Slow	0.00	(Reference)				0.00	(Reference)			
Women										
Rapid	2.20	(0.12–4.27)	1.05	2.09	0.038	5.31	(− 0.56–11.18)	2.97	1.79	0.076
Moderate	0.03	(− 1.84–1.91)	0.95	0.04	0.971	1.80	(− 3.51–7.11)	2.69	0.67	0.504
Slow	0.00	(Reference)				0.00	(Reference)			

Adjusted for age, the frequency of current alcohol consumption, current smoking, skipping breakfast, eating snacks, having dinner within 2 h of bedtime, physical activity, and depression.

*BMI* body mass index, *CI* confidence interval, *WC* waist circumference.

## Discussion

In the present cross-sectional study, we aimed to evaluate the relationships of eating speed with abdominal fat and adiponectin in adult men and women. Specifically, we evaluated the relationships of self-reported eating speed with the VFT and SFT areas, measured using CT, and with the plasma adiponectin concentration. We found that rapid eating was associated with larger VFT and SFT areas and lower adiponectin concentration in men, which is partially consistent with the hypothesis. However, in women, rapid eating was found to be associated only with BMI; there were no significant relationships of eating speed with abdominal fat mass or adiponectin concentration, which is a result that is not consistent with the hypothesis. These findings suggest that there is no difference in the association of rapid eating with VFT and SFT areas.

We found that rapid eating was associated with the larger VFT and SFT areas in men but was also associated with higher BMI and larger WC. These results are consistent with those of previous anthropometric studies, which showed that rapid eating is associated with obesity and overweight^3,4^. In previous CT and MRI-based studies in which VFT and SFT components were separately measured, contrasting results were obtained. Iwasaki et al.^17^ and Mochizuki et al.^18^ reported that rapid eating is associated with larger VFT but not SFT area in adults, whereas Fogel et al*.*^19^ showed that rapid eating is associated with larger VFT and SFT volumes in children. These differing findings may be explained by the varied characteristics of the participants in the studies. For example, the male participants in the present study were older than those in the studies of Iwasaki et al*.*^17^ and Mochizuki et al.^18^. Low testosterone and osteocalcin concentrations, which are known to be associated with high adiposity^30–32^, and low muscle mass and basal metabolism may modulate the effects of rapid eating on SFT, as well as VFT, in older participants.

The mechanism underlying the relationship between rapid eating and abdominal fat accumulation remains unclear. One possibility is that rapid eating may cause insufficient satiety, leading to overeating and a higher total energy intake^33^, possibly because brief periods of sensory exposure provide insufficient cues for satiety^34^ and are associated with lower secretion of gastrointestinal hormones that control this^35^. A previous laboratory study showed that rapid eating induces lower postprandial energy expenditure^23^, which may contribute to fat accumulation, even in the absence of overeating.

Unlike in men, eating speed was shown not to be associated with either the VFT or the SFT areas in women. Instead, it was found to be associated only with BMI. BMI is a measure of body mass that is adjusted for height, and therefore does not depend solely on abdominal fat mass. Adipose tissue is more likely to accumulate in the abdomen in men, while women more frequently accumulate fat in the gluteofemoral region. Thus, rapid eating may be associated with fat accumulation in the gluteofemoral region, rather than in the abdomen, in women.

The present findings in women are not consistent with the results of previous CT and MRI-based studies, which have shown that rapid eating is associated with larger abdominal fat depots in adult men and women^17^, and also in boys and girls^19^. Although some studies using anthropometric measures have shown that rapid eating is associated with larger abdominal fat depots in men and women (e.g. Otsuka et al.^3^; Wuren et al.^4^), others have shown that a relationship between rapid eating and higher BMI^6,36^ exists in men, but not in women. In contrast to the consistent results obtained for men, contradictory results have been obtained for women. Women have been shown to prefer less caloric foods^37^ and are more likely to engage in dieting behavior to improve their physical appearance^38^. These eating behaviors may attenuate the effects of eating speed on abdominal fat mass. Alternatively, because estrogen and progesterone regulate hunger and appetite^39^, and the participants in the present study were relatively old, low estrogen and progesterone levels or an imbalance of the two may explain the lack of a relationship between rapid eating and abdominal fat mass in these older women.

We have also shown that rapid eating is associated with low adiponectin concentration in men. To the best of our knowledge, this is the first study to show a relationship between eating speed and adiponectin. However, the finding is consistent with previous findings that rapid eating is associated with the levels of metabolic markers associated with obesity and overweight, such as low HDL-cholesterol concentration, high TG concentration, and high alanine aminotransferase (ALT) activity^36,40,41^. Rapid eating would be expected to be associated with a lower concentration of adiponectin, at least partly because of its effect to cause the accumulation of abdominal fat. In women, we found no relationship between eating speed and adiponectin concentration. This may be because eating speed is not related to abdominal fat mass in women. Previous studies have demonstrated that adiponectin concentration is related to smoking status^42^, exercise, diet^43^, and coffee consumption^44^. The results of the present study extend the list of lifestyle factors, which are associated with adiponectin concentration, to include eating speed.

Eating speed can be controllable, and the reduction of eating speed represents a potential intervention strategy for the treatment of obesity and overweight. Previous intervention studies have shown that reducing eating speed by means of chewing food in the mouth at least 30 times reduces BMI in patients with obesity^13^. The findings of the present study may imply that reducing eating speed may reduce VFT and SFT mass and increase adiponectin concentration, particularly in men. Future intervention studies should test these possibilities.

The present study had several limitations. Eating speed was assessed using a self-reported, single-item questionnaire. This method has frequently been used in previous studies on the relationships between eating speed and obesity and related diseases, and has been validated using objective measures of eating speed^24,25^. However, previous research has reported a lack of high agreement between eating speed assessed using this self-reported questionnaire and objectively measured eating speed at an individual level^45^. Another single-item eating speed questionnaire^46^ measures the time taken to consume meals; respondents are asked “How long does it take you to eat a meal?” for each of breakfast, lunch, and dinner. This latter questionnaire may reduce the bias caused by subjective self-evaluation of eating speed. The subscale “Slowness in Eating,” which comprises four items on the Adult Eating Behavior Questionnaire^47^ has also been used to evaluate eating speed. This self-reported questionnaire is a psychometrically valid and reliable measure of eating behavior. In the laboratory setting, eating speed has been assessed using various objective measures, including continuous weighing of foods using an electronic balance^48^ and overall food consumption time^13,49^. The types and amounts of test foods vary among studies^13,49^. There is no standardized protocol for assessing eating speed^45^, which limits comparability across studies. Future studies should aim to develop a standardized protocol and use it to attempt to replicate the present findings.

In the present study, the sample of women (*n* = 184) was smaller than that of men (*n* = 528). However, the number of female participants in this study is comparable to the number of female participants in some previous studies^17,50^. Nevertheless, the smaller female sample size may have reduced the statistical power to detect an association between women’s eating speed and abdominal fat mass and adiponectin. These associations in women were similar to those in men, although they were not significant. Thus, the lack of significant results for women mean that the findings should be interpreted with caution. Furthermore, it was cross-sectional in nature, and thus conclusions cannot be drawn regarding causal relationships of eating speed with abdominal fat mass and adiponectin concentration. We did not have information regarding the type or amount of food consumed, and therefore we could not test the effects of the diet consumed on the relationship between eating speed and abdominal fat mass. Finally, the study sample was limited to workers in agriculture and related industries and their families living in a rural area of Japan, which may reduce the external validity of the findings.

In conclusion, in the present study, we have shown that rapid eating is associated with larger VFT and SFT areas and lower adiponectin concentration in men. In contrast, while rapid eating was found to be associated only with BMI, eating speed was found not to be associated with abdominal fat mass or adiponectin concentration in women. The present findings are consistent with previous findings that rapid eating is associated with the development of obesity and overweight, and imply that adult men may benefit more from reducing their eating speed.

## Data Availability

The datasets generated during and/or analyzed during the current study are not publicly available due to participants’ confidentiality concerns but are available from the corresponding author on reasonable request.
